# Acoustic energy harvesting using phononic crystal fiber with conical input

**DOI:** 10.1038/s41598-024-59528-z

**Published:** 2024-05-29

**Authors:** Farzaneh Motaei, Ali Bahrami

**Affiliations:** Optoelectronics and Nanophotonics Research Lab. (ONRL), Faculty of Electrical Engineering, Sahand University of Technology, Tabriz, Iran

**Keywords:** Phononic crystal fiber, Environmental acoustic waves, Coupler, Conical input, Structure of solids and liquids, Acoustics

## Abstract

In this paper, a novel phononic crystal fiber with conical input is introduced for coupling environmental acoustic waves to the fiber core. Environmental acoustic waves can be focused and coupled to the core through conical input. In the first step, a cone shape coupler has been considered for coupling the incident acoustic waves to the core region. This initial idea has a significant acoustic energy loss. This disadvantage encourages us to design a new phononic crystal fiber without it. Designed structure includes a phononic crystal fiber with conical input meaning solid rods have been shaped in conical way at the input section of fiber. By using this structure, environmental acoustic waves can be properly coupled to the core region of the fiber. Acoustic wave leakage to outside of the Phononic crystal fiber has been extremely decreased in comparison with initial coupler. Experimental results indicate that environmental acoustic waves can be focused and coupled to the core region by phononic crystal fiber with conical input.

## Introduction

Phononic crystals are artificially constructed by embedding an array of inclusions in a host material in which physical properties of these two phases are highly different. According to the material phases, phononic crystals are designed in three types: solid–solid, fluid–fluid, and solid–fluid structures. From the inclusion periodicity point of view, it can be arranged in one, two, and three dimensions. For more than ten years, phononic crystals have attracted the attention of many researchers due to their ability to control and manipulate acoustic waves. The most important ability of these structures is to confine the acoustic waves with high quality. This capability emanates from bandgap appearance in dispersion curves of periodic structure. Repetition of inclusions in a host material with high acoustic contrast between them makes one or more forbidden frequency regions in the dispersion curves called a phononic bandgap. Any structural variation in periodic structure, such as defects, produces excessive pass bands or defect modes. In order to achieve the acoustic wave confinement, defect modes should be placed in phononic bandgap. In this way, acoustic waves with frequency of defect mode can be confined in defect region of structure^1^. There are many acoustic devices which operate based on this concept such as waveguides^2^, filters^3^, demultiplexers^4^, switches^5^, sensors^6,7^, energy harvesters^8,9^, etc.

Recently, a novel configuration of phononic crystals named as phononic crystal fiber has been presented in our previous works^9,10^. Structural setup of the phononic crystal fibers are the same as photonic crystal fibers, both of them have been fabricated by running an array of rods along fiber length. The high capability of phononic crystal fiber to confine acoustic wave has been discussed and compared with the other previous similar waveguides in Ref.^10^. Since phononic crystal fibers have an acceptable performance to confine and guide acoustic waves, it can be sensed that this sonic structure will play important roles in many applications such as sonar, sensing, acoustic wave focusing, energy harvesting and etc. in near future.

In our previous work, phononic crystal fiber has been introduced to energy harvesting application of factory noises. Details of advantages and abilities of phononic crystal fiber energy harvester have been discussed and compared with the other works in it^9^. Using phononic crystal fiber energy harvesters have two advantages: high confinement capability and guiding ambient sonic waves to the energy converting station through fiber core. Although the phononic crystal fiber has a high confinement ability, this property is not enough for energy harvesting goal. High confinement is the inherent capability of phononic crystal fibers. In fact, it is a quality index to estimate guiding performance with low radial loss. In the other words, a structure with very high confinement can transmit the incident wave without any change in its amplitude. Besides the high confinement, focusing and coupling environmental waves to realize energy harvesting with high electrical output are necessary. Without any coupling device, the generated output voltage of phononic crystal fiber is almost equal to generated voltage in the bare case. Thus, phononic crystal fibers also require a coupler with a phononic crystal.

In this paper, we focus on designing a new device to couple and guide environmental acoustic waves in phononic crystal fibers. The novelty of this work is environmental acoustic waves coupling capability to the fiber core without using any external couplers such as acoustic lenses. In 2017, a Luneburg lens based on phononic crystal has been designed in which acoustic waves can concentrate at the local point with multiplication value equals to 2.5 times. Also, the harvested voltage from this structure is 2.5 times in comparison with bare case^11^. Also, in 2019, a 3D-printed phononic crystal lens has been presented to focus acoustic waves with multiplication almost equals to 1.8 times^12^. The ability of conical input and lenses to focus the acoustic waves is similar, because in both structures, acoustic waves are concentrated through an almost conical path. Designing the phononic crystal lenses requires more inclusions and larger space than sonic fiber. Also, designing a conical input is simpler than designing and formulating an acoustic lens. In the case of using a lens, it is necessary to match the acoustic impedance between the wave propagation medium in both structures (lens and fiber). Therefore, using an external concentrator (such as lens) requires more cost and time in comparison with sonic fiber with conical input. In addition, the fabrication of the lens structure will have a fabrication tolerance that will increase the total tolerance of the lens and fiber assembly. Hence, using the conical input technique in the phononic crystal fiber instead of external acoustic lenses reduces time, cost, and fabrication tolerance.

In the first step, we have taken inspiration from a taper waveguide to design an initial fiber coupler. A discussion about disadvantages of this coupler will be done. Then, regarding to the last discussions and disadvantages of cone shape coupler, a new phononic crystal fiber will be presented which has been enabled to couple environmental waves into fiber core.

Next sections are as follows: phononic crystal fiber coupler in section "Phononic crystal fiber coupler", phononic crystal fiber with conical input in section "Phononic crystal fiber with conical input", discussions in section "Discussions", and conclusions in section "Conclusions".

## Phononic crystal fiber coupler

Sonic fibers, like optical fibers, require coupling components to couple incident waves to the core. Some applications need to transmit acoustic waves with high intensity such as electrical energy harvesting. Recently, some investigations about acoustic wave focusing by acoustic lenses have been done^8,13^. However, it is not economical because design and fabrication of acoustic lenses are relatively complex. Therefore, we have present a new, simple, and efficient structure to couple environmental acoustic waves to a fiber core. In the first step, we were inspired from taper waveguide used in 2D photonic crystal structure to design the acoustic coupler for phononic crystal fiber^14^. Since sonic fibers have a circle cross-section input, taper waveguide should be promoted to cone shape coupler. In the next subsection, coupling capability of cone shape coupler is investigated. Then, a phononic crystal fiber equipped with designed cone shape coupler will be presented.

### Cone shape coupler

Here, we prove the coupling capability of a cone shape coupler. This initial step introduces us to how the cone shape coupler works to focus and couple the environmental acoustic waves to the waveguide. According to results of the initial structure, we can decide about ability and disability of it as a coupler for phononic crystal fiber. Total schematics and simulation results are available in following.

#### Design procedure

**Figure 1:**
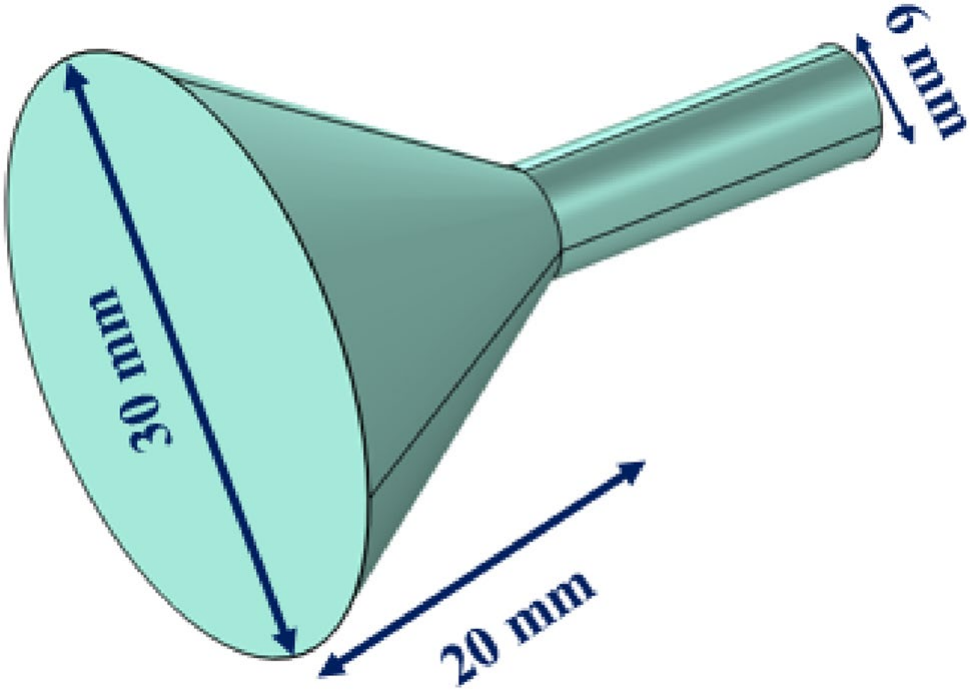
Schematics of a cone shape coupler along with simple waveguide.

This subsection presents a Poly Methyl Methacrylate (PMMA) cone shape coupler along with simple waveguide as Fig. 1.

According to the Fig. 1, left and right diameters of coupler and its length are equal to 30 mm, 6 mm, and 20 mm, respectively. The material of the structure is PMMA which its elastic parameters are $\rho = {1190}\,{\text{kg}}\,{\text{m}}^{{-3}}$, $E = 3\,{\text{GPa}}$, and $\nu = 0.35$. Proposed device is a solid structure so elastic wave propagation relation must be considered as following^1^:

1 $$ T_{ij} ({\mathbf{r}},t) = c_{ijkl} ({\mathbf{r}})u_{k,l} ({\mathbf{r}},t) $$

2 $$ T_{ij,j} ({\mathbf{r}},t) = \rho ({\mathbf{r}})\frac{{\partial^{2} u_{i} ({\mathbf{r}},t)}}{{\partial t^{2} }} $$

where *T*_*ij*_ and *u*_*i*_ are stress tensor and displacement. The other parameters, $\rho$ and *c*_*ijkl*_ are mass density and elastic constants. Indices *i*, *j*, *k*, and *l* define values for three space components. Derivation concept has been defined by a comma (e.g., $u_{k,l} = \frac{{\partial u_{k} }}{{\partial x_{l} }}$), and summation over repeated indices is implied (e.g., $T_{ij,j} = \sum\nolimits_{j = 1}^{3} {\frac{{\partial T_{ij} }}{{\partial x_{j} }}}$). By combination of two above relations, we have^1^:

3 $$ (c_{ijkl} ({\mathbf{r}})u_{k,l} ({\mathbf{r}},t))_{,j} = \rho ({\mathbf{r}})\frac{{\partial^{2} u_{i} ({\mathbf{r}},t)}}{{\partial t^{2} }} $$

which it is the main relation for solid structures modeling. Elastic wave propagation relation should be solved, so finite element numerical method has been utilized for this purpose.

#### Simulation results

**Figure 2:**
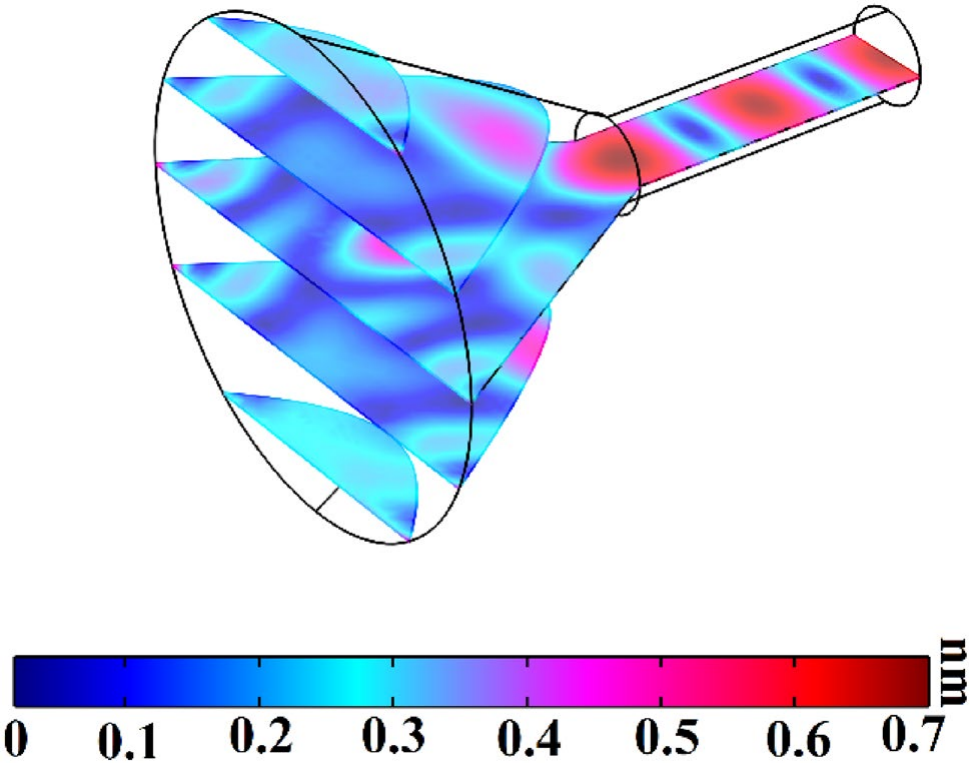
Coupling capability of cone shape coupler.

Simulation results corresponded to the cone shape coupler (Fig. 1) has been calculated by finite element method software in frequency of 82.4 kHz and has been illustrated in Fig. 2.

**Figure 3:**
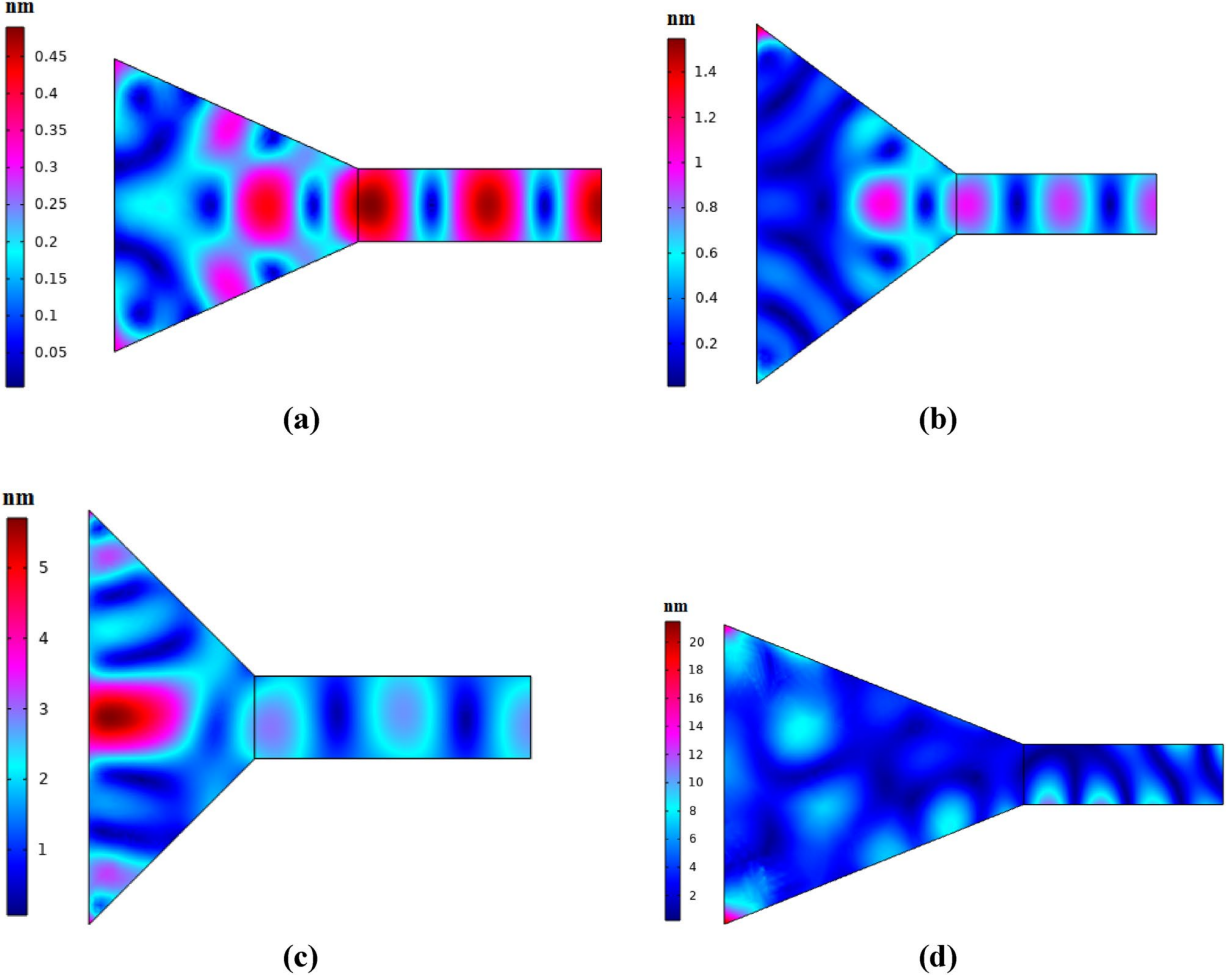
Coupling capability of cone shape coupler with (**a**) inlet radius = 12 mm and coupler length = 20 mm, (**b**) inlet radius = 18 mm and coupler length = 20 mm, (**c**) inlet radius = 15 mm and coupler length = 12 mm, and (**d**) inlet radius = 15 mm and coupler length = 30 mm.

As can be seen from Fig. 2, cone shape coupler can concentrate the incoming waves almost 2.3 times. Input width and coupler length are two important parameters for acoustic wave concentrating. Figure 3 shows different values of these two parameters and their effects on the acoustic wave concentration.

**Figure 4:**
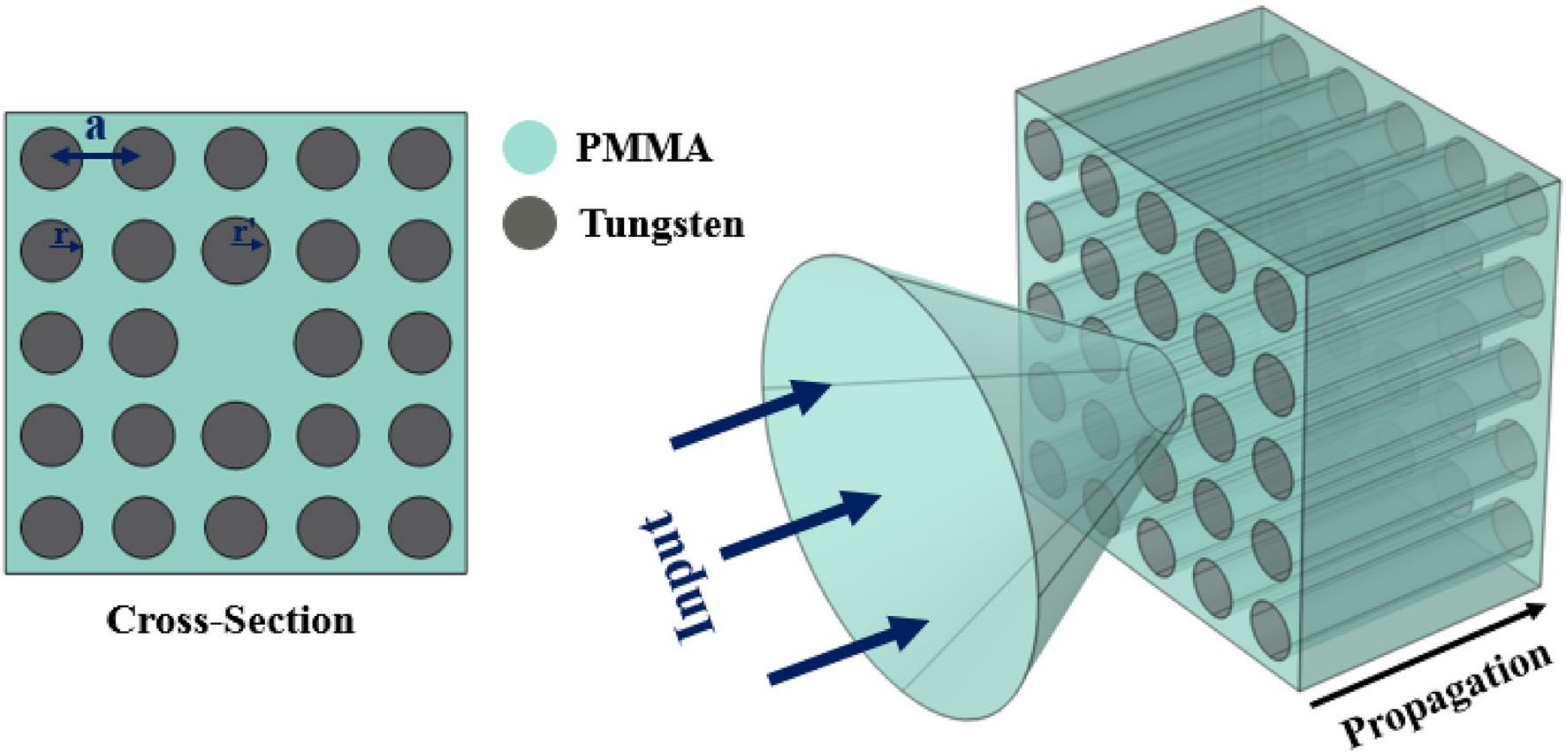
Schematics of phononic crystal fiber with cone shape coupler.

According to the Fig. 3a, concentration is almost 2.2 times. Decreasing the input area decreases acoustic waves entering to the coupler, so concentration decreases. By appropriate increasing the input area (Fig. 3b), concentration increases almost 3 times. According to the Fig. 3c, it is clear that decreasing the coupler length makes significant part of acoustic waves losses to the outside. The high slope of the wall leads to the outside losses. In Fig. 3d, concentration has not occurred. The working frequency for all cases is 82.4 kHz. Dimensions of coupler in Fig. 1 has been selected according to resonant frequency of next structure (Fig. 4), because this coupler will attach to the phononic crystal fiber.

We can conclude from this part that environmental acoustic waves can be concentrated by cone shape coupler. Therefore, this coupler can be attached to phononic crystal fiber which has been done in next subsection.

### Phononic crystal fiber with cone shape coupler

Here, the designed cone shape coupler has been attached to the phononic crystal fiber. Its design procedure and simulation results are available in the following subsection.

#### Design procedure

It is clear that fiber structures include two well-known regions; core and clad. In crystal-based fibers, photonic/phononic crystals operate as cladding. In order to achieve high quality confinement, natural frequency of core should be placed in phononic crystal bandgap. In this structure, tungsten rods with square arrangement have been embedded in a PMMA host. Schematics of designed structure with coupler have been depicted in Fig. 4.

According to Fig. 4, radius of tungsten rods in surroundings is $r = 2\,{\text{mm}}$, and distance between centers of rods is $a = 6\,{\text{mm}}$. The fiber core has been formed by removing a single central rod. In order to optimize resonance frequency of core, it is surrounded by four tungsten rods with radii equal to $r^{\prime} = 2.2\,{\text{mm}}$. Mechanical properties of tungsten are $\rho = \,19{,}350\,{\text{kg}}\,{\text{m}}^{-3}$, $E = 411\,{\text{GPa}}$, and $\nu = 0.28$.

#### Simulation results

**Figure 5:**
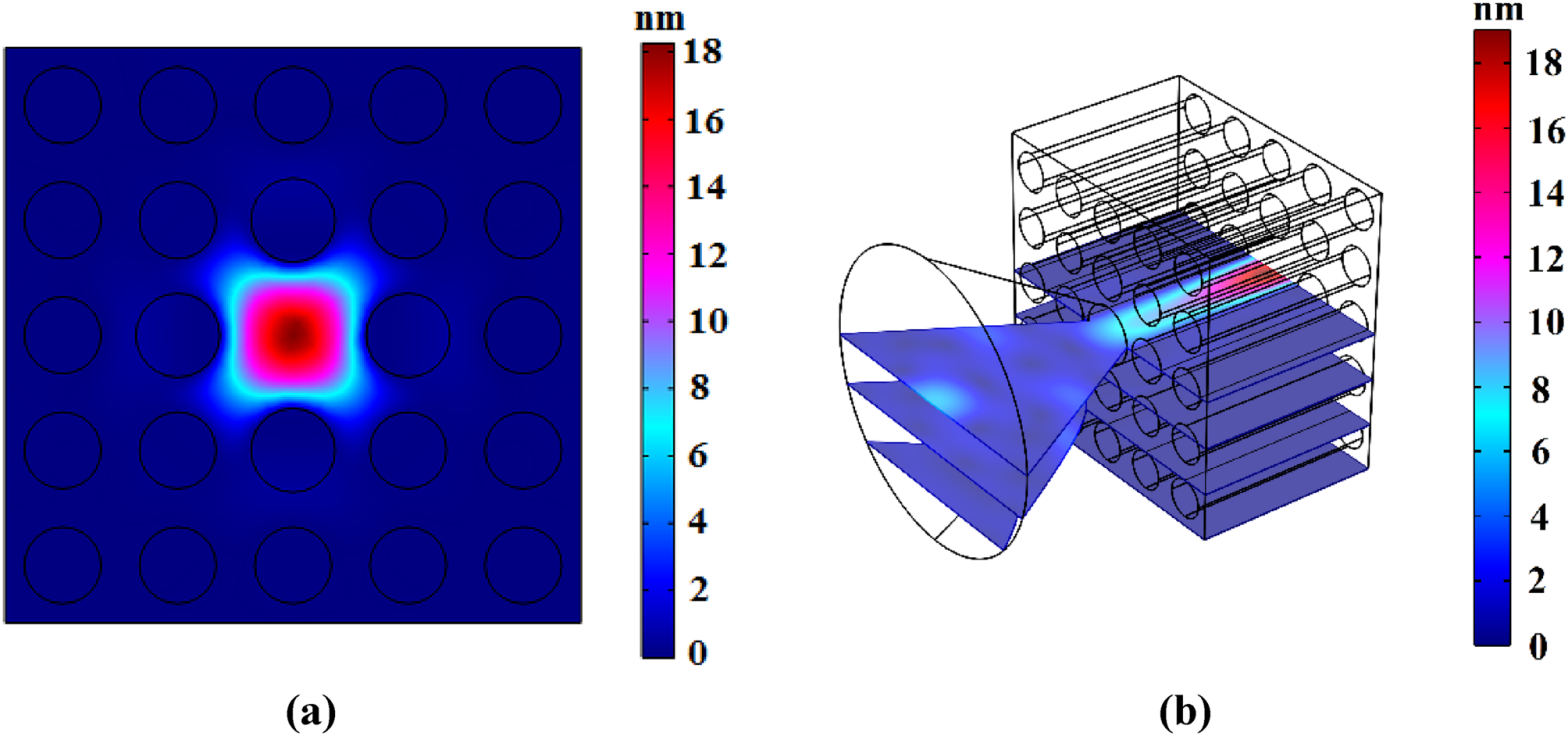
Displacement field in frequency of 82.4 kHz; (**a**) cross-sectional and (**b**) longitudinal views.

This subsection has been presented to report simulation results of designed elastic fiber with cone shape coupler. At a frequency of 82.4 kHz, acoustic wave confinement in core region with fiber length of 20 mm has been shown as Fig. 5.

Cross-sectional and longitudinal views of displacement field are illustrated in Fig. 5a and b, respectively. Also, it can be seen that phononic crystal fiber shown in Fig. 4, confines applied wave with frequency of 82.4 kHz, thoroughly. In this step, an acoustic fiber with very low radial loss in comparison with the simple PMMA waveguide has been designed.

Comparison between results of Figs. 2 and 5b.  shows that existence of phononic crystal waveguide instead of simple PMMA waveguide makes wave amplitude increases from 0.7 to 18 nm. This phenomenon is the result of wave leakage to outside of the structure. Wave reflection of the coupler and simple waveguide boundaries is much lower than the reflection capability of the phononic crystal. Although the proposed phononic crystal fiber with cone shape coupler reduces the acoustic wave leakage to outside, high losses exist in the coupler part of the fiber structure. Therefore, we can conclude that cone shape coupler is not a perfect structure to couple environmental acoustic waves into core region. In the next section, we attempt to introduce a new phononic crystal fiber with coupling capability for environmental acoustic waves.

## Phononic crystal fiber with conical input

According to the previous section, it is clear that phononic crystals have a high ability in acoustic wave confinement. The simple cone shape coupler was losing a significant part of the acoustic waves to the outside, which is not an ignorable disadvantage. Therefore, we introduce a new phononic crystal fiber with coupling capability without an external coupler in the current section. This structure can reduce the acoustic wave leakage of the cone shape coupler to the outside. Design procedure, simulation results, and experimental details of phononic crystal fiber with conical input are available in following subsections, respectively.

### Design procedure

**Figure 6:**
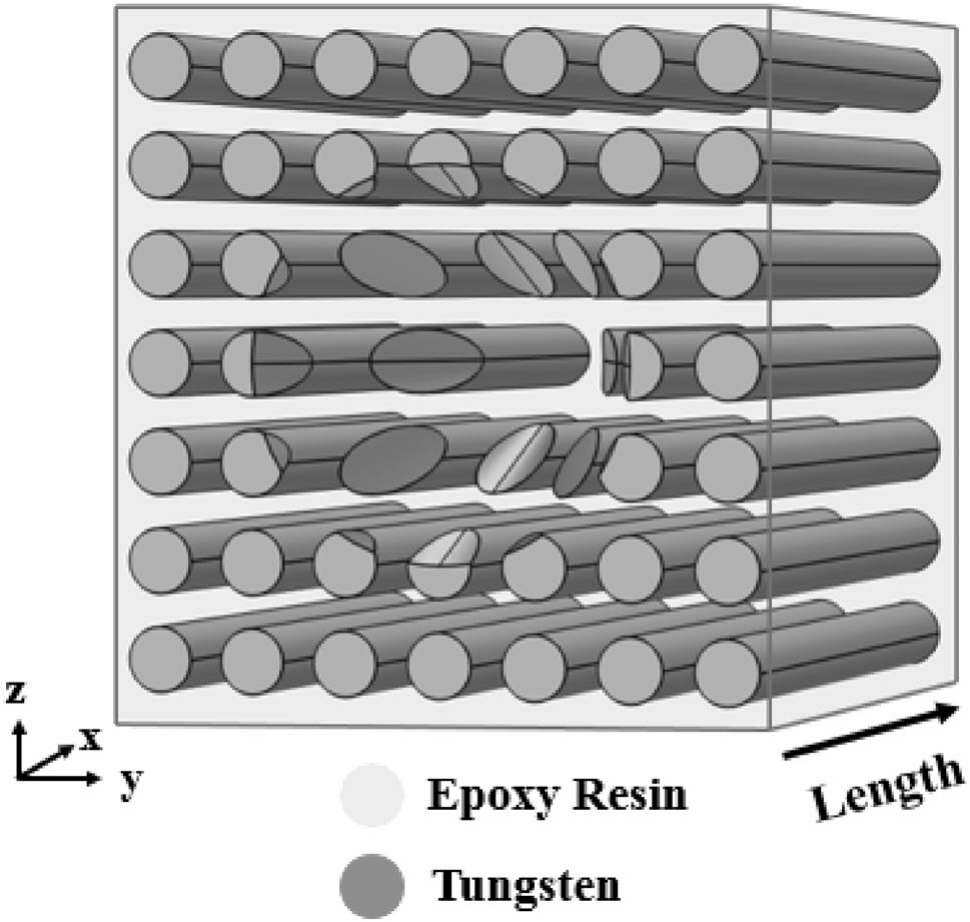
Phononic crystal fiber with conical input.

In this step, design procedure of phononic crystal fiber with acoustic wave coupling capability depicted in Fig. 6 has been introduced.

Phononic crystal fiber shown in Fig. 6 has been formed by tungsten rods in epoxy resin host with square configuration. In order to couple the environmental acoustic waves to core region, tungsten rods in input part of fiber have been shaped in conical way. Radii of all rods are equal to 2 mm, and the lattice constant is equal to 6 mm. Mechanical properties of epoxy resin are $\rho = \,1250\,kg.m^{ - 3}$, $E = 3.5\,GPa$, and $\nu = 0.33$. Core region has been formed by removing a single central rod. Especial design of fiber input helps to concentrate environmental acoustic waves and couple to core region. The phononic crystal in surrounding of conical input reduces the wave leakage to the outside. This is the most important advantage of this coupling technique compared with previous structure.

### Simulation results

**Figure 7:**
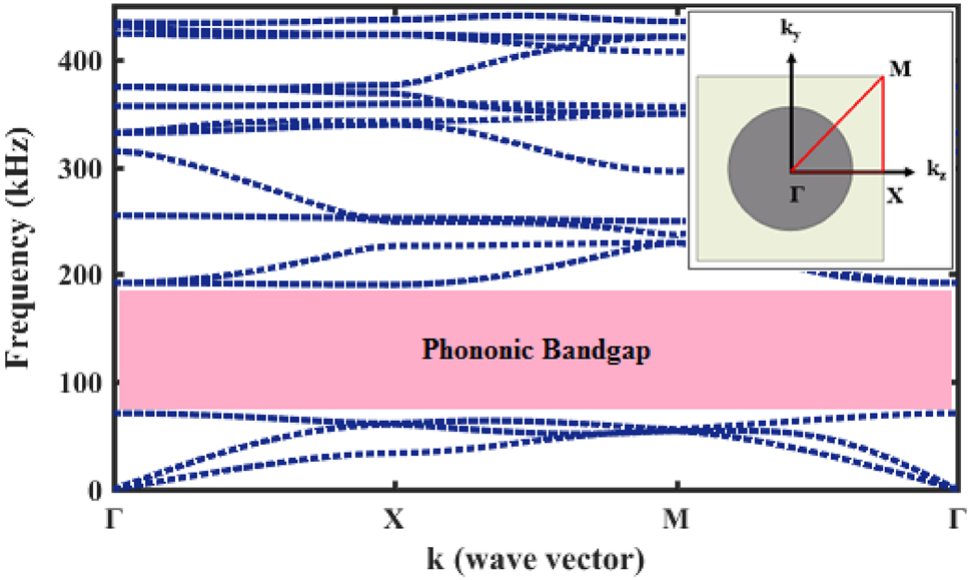
Band diagram of 2D cross-section of perfect phononic crystal.

The fundamental equation for describing of elastic wave propagation, Eq. 3, has been solved by finite element numerical method. The role of phononic crystal in surroundings of core is to reduce the radial leakages of acoustic waves. In this way, band diagram of 2D cross-section of perfect phononic crystal has been illustrated in Fig. 7.

**Figure 8:**
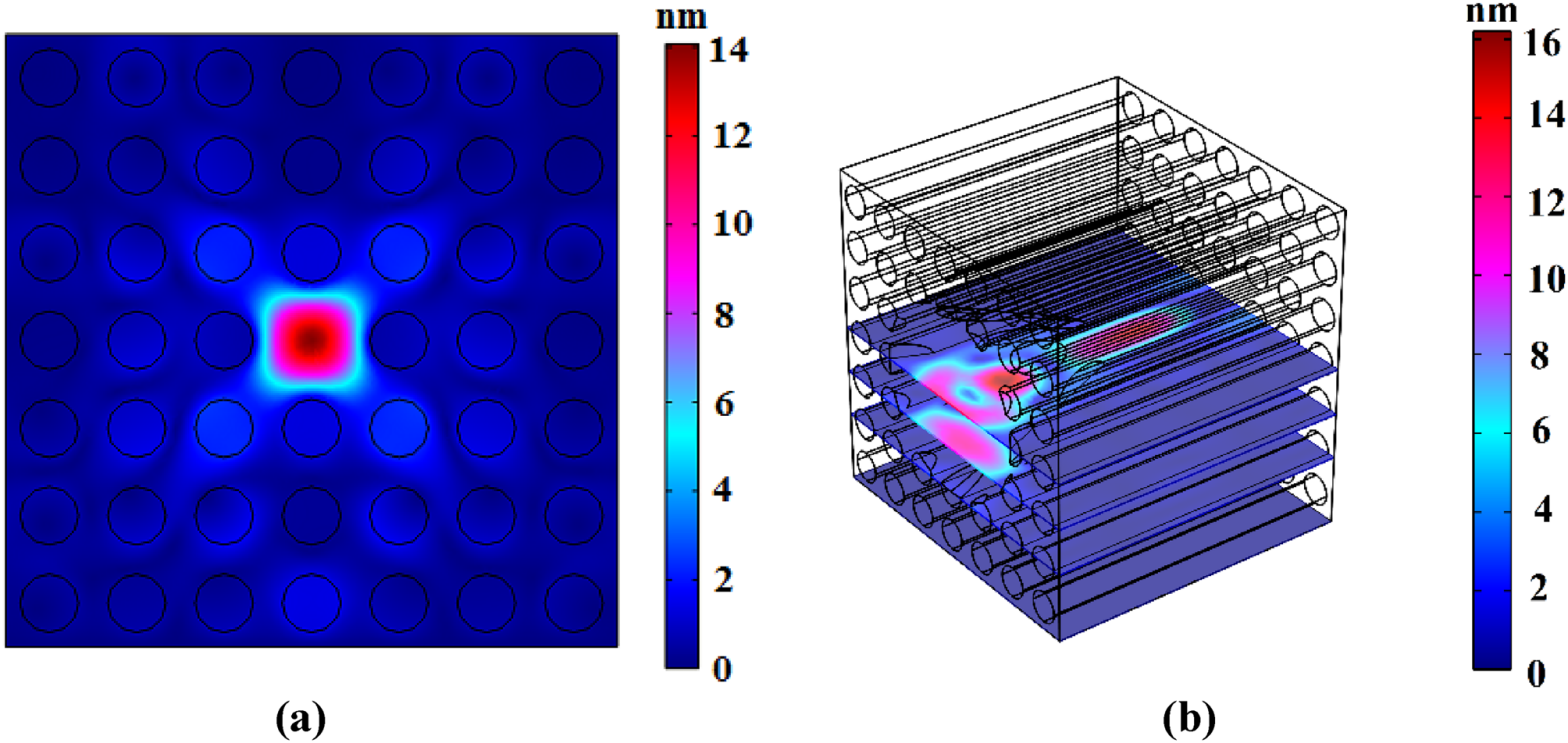
Displacement field in frequency of 91.4 kHz; (**a**) cross-sectional and (**b**) longitudinal views.

It can be seen from Fig. 7 that acoustic waves which are applied to the lateral of tungsten rods have been blocked in frequency range of 70 kHz to 190 kHz. The best acoustic wave confining will be realized when the frequency of the input wave is in the blocking range and equal to the core resonant frequency, simultaneously. The resonant frequency of fiber core is equal to 91.4 kHz. Figure 8 depicts coupling and confining incident acoustic wave with frequency of 91.4 kHz in proposed phononic crystal fiber.

**Figure 9:**
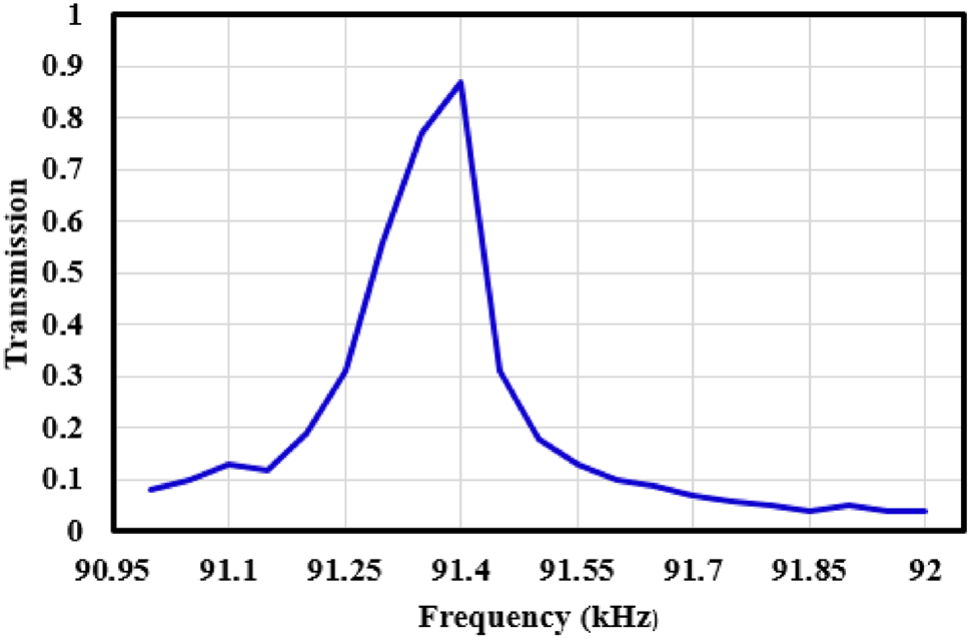
Transmission spectrum of presented phononic crystal fiber around frequency of 91.4 kHz.

According to Fig. 8b, it is clear that confinement of incident waves in conical part of fiber has been increased to 11 nm in comparison with Fig. 5b. Also, incident waves can couple to the core in conical way. Frequency of incident wave is equal to 91.4 kHz. Total fiber length is equal to 40 mm, while the waveguide length without conical input is 20 mm. Transmission spectrum of presented phononic crystal fiber around frequency of 91.4 kHz is available in Fig. 9.

According the Fig. 9, maximum confinement and transmission is occurred in frequency of 91.4 kHz. Designed phononic crystal fiber illustrated in Fig. 6 has been experimentally tested in followings.

### Fabrication process and experimental results

**Figure 10:**
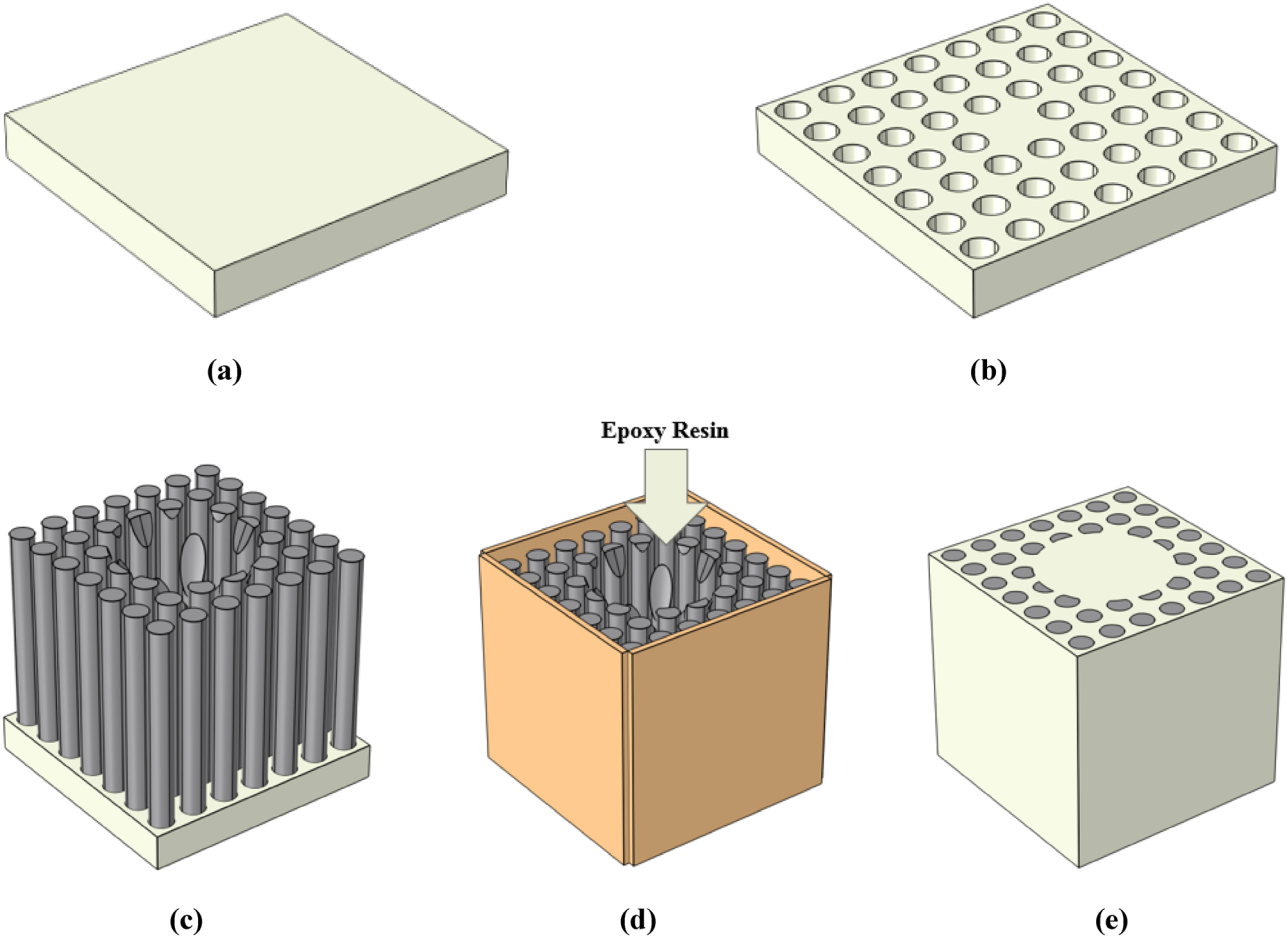
Fabrication process of phononic crystal fiber; (**a**) preparation of an epoxy resin plate, (**b**) epoxy resin plate perforation to tungsten rods embedding, (**c**) tungsten rods embedding in epoxy resin plate, (**d**) molding around the structure to add epoxy resin in liquid phase, and (**e**) removing the mold after epoxy resin cure time.

Confinement capability of the new acoustic fiber has been theoretically investigated in previous subsection. In order to investigate the proposed phononic crystal fiber in practice, it has been fabricated and experimental results will be reported in this step. Fabrication process of this fiber has been explained by Fig. 10.

**Figure 11:**
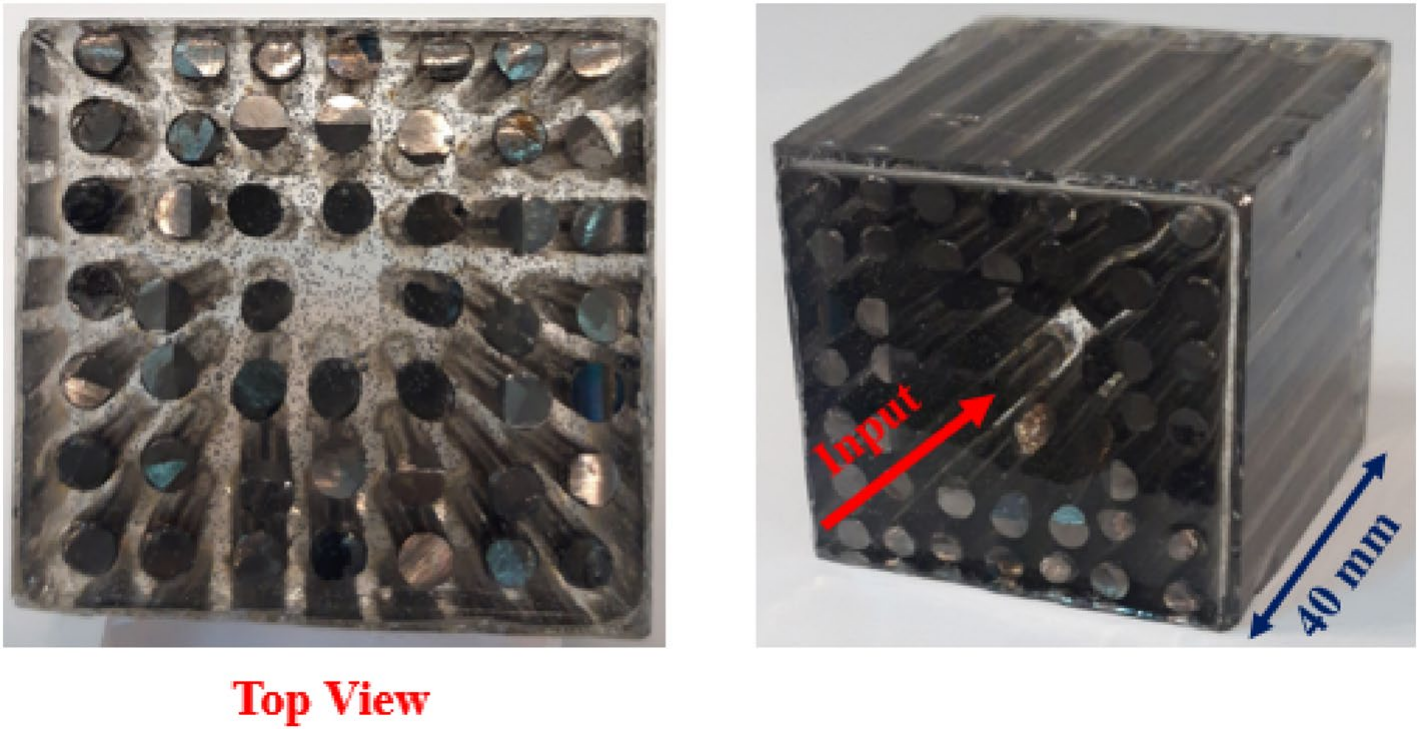
Phononic crystal fiber with conical input prototype.

According to Fig. 10a and b, an epoxy resin stand has been prepared to hold the tungsten rods in air. In next step, tungsten rods have been embedded in epoxy resin stand. Then, surroundings of structure are molded and epoxy resin in liquid phase is spilled. After epoxy resin cure time, the mold was removed and phononic crystal fiber was prepared. Figure 11 presents fabricated phononic crystal fiber with conical input prototype.

**Figure 12:**
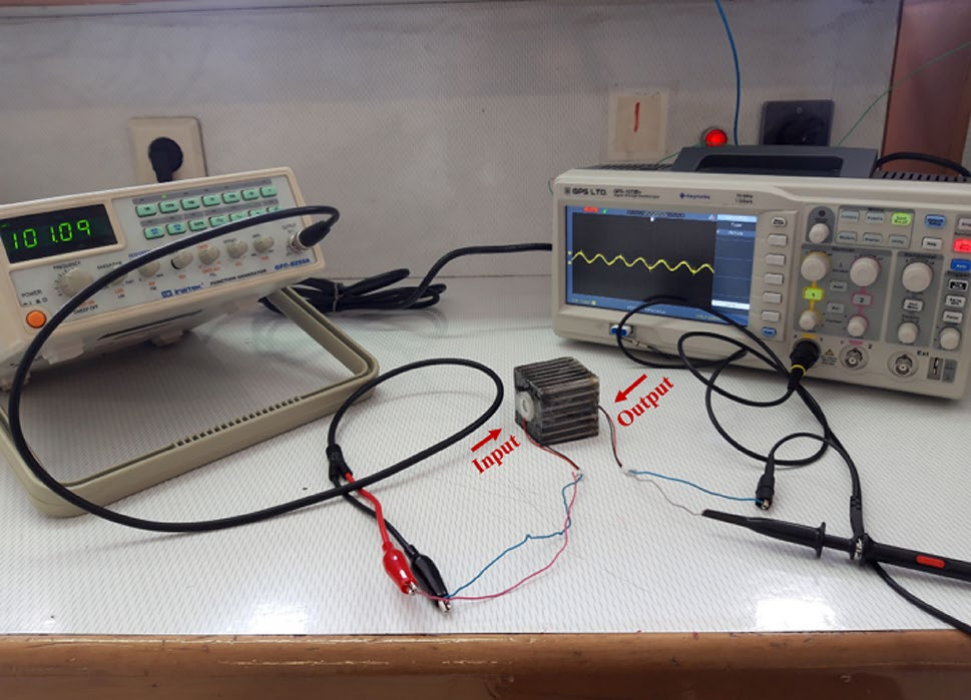
Experimental setup assembled to testing the fiber prototype.

Three-dimensional and top views of fabricated phononic crystal fiber are seen in Fig. 11. The host material is epoxy resin and tungsten rods have been configured in conical shape at input part of structure. Figure 12 presents the experimental setup assembled to testing the fiber prototype.

**Figure 13:**
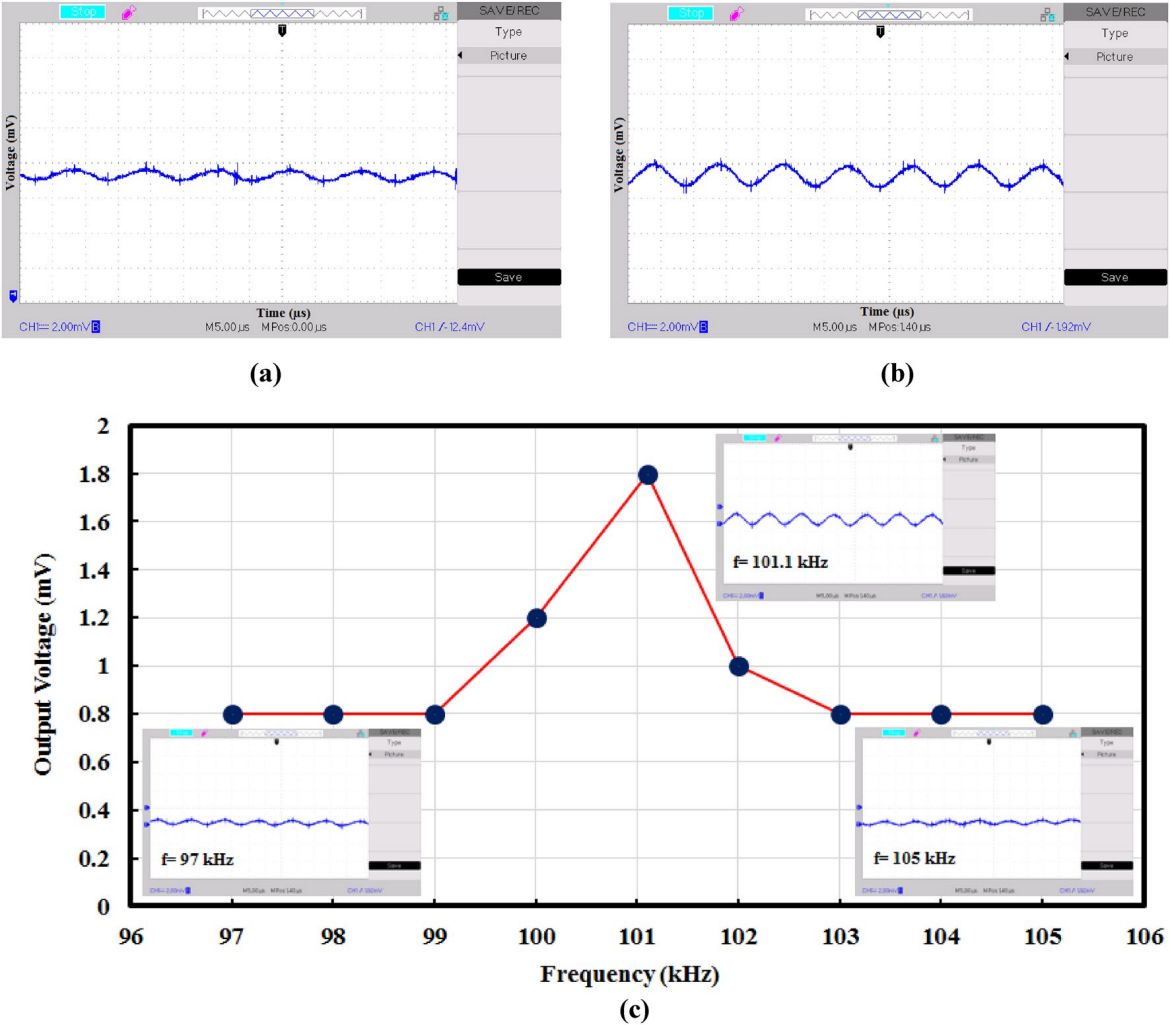
Experimental results; (**a**) bare case, (**b**) phononic crystal fiber case with frequency of 101.1 kHz, and (**c**) recorded output voltage of phononic crystal fiber in different frequencies.

Regarding to Fig. 12, an experimental configuration has been considered including a piezoelectric transducer and function generator to generate an acoustic wave. Piezoelectric transducer is a disc-type lead zirconate titanate (PZT) with resonant frequency of 108 kHz, and it has been directly attached to the conical input of fiber. The input voltage to drive the piezoelectric is equal to 15 V. Output data has been recorded by a same piezoelectric and oscilloscope. Output piezoelectric has been directly attached to the end of fiber core. Recorded experimental results in bare case and phononic crystal fiber case have been presented in Fig. 13.

It can be seen from Fig. 13a that the recorded output voltage is equal to 0.8 mV in bare case. By applying the acoustic waves to phononic crystal fiber, the output voltage can increase to 1.8 mV in frequency of 101.1 kHz. The values of Volt/div and Time/div of oscilloscope are equal to 2 mV and 5 µs, respectively. This effect appears due to acoustic wave focusing by conical input. Resonant frequency of phononic crystal fiber is different from simulation result. This difference is due to existence of the PZT and fabrication tolerance. It is clear that by increasing the conical input area, the acoustic wave coupling and consequently the output voltage increases. If there is no loss to the outside, acoustic wave concentration is equal to A_input_/A_core_. Acoustic waves that hit the input area of coupler will be concentrated at the end area of taper coupler (or core area). The output voltage multiplication is equal to the ratio of input area to core area. Therefore, we can conclude that phononic crystal fiber with conical input properly acts in practice space.

## Discussions

**Figure 14:**
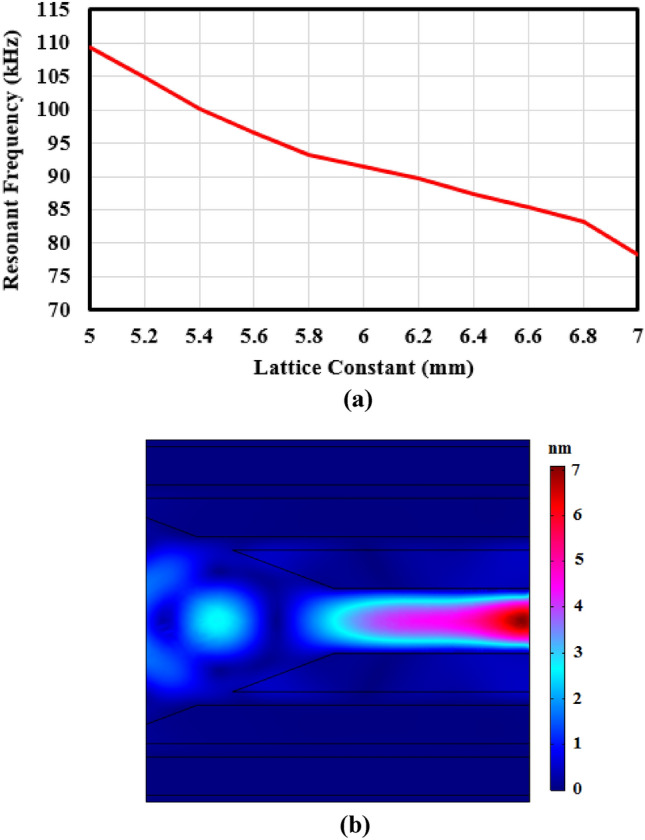
(**a**) Resonant frequency of fiber core versus lattice constant verifications, and (**b**) displacement field with lattice constant equals to 5.4 mm and incident frequency of 101 kHz.

In this section, we have tried to explain difference between simulation and experimental results. Resonant frequency is equal to 91.4 kHz in simulation case, but it has shifted to 101.1 kHz in experimental measuring. One of the most important reason for this resonant frequency shift to high range is deduction of lattice constant in fabrication process. Therefore, effect of lattice constant verification has been explored in simulation way which related results have been depicted in Fig. 14.

It can be seen from Fig. 14a that by decreasing the lattice constant resonant frequency of phononic crystal fiber is shifted to higher frequencies. According to the experimental data and Fig. 14, we can conclude that there is a lattice constant fabrication tolerance about 0.6 mm. Figure 14b shows confinement of incident wave with frequency of 101 kHz in phononic crystal fiber with lattice constant of 5.4 mm.

## Conclusions

In this investigation, it has been attempted to introduce a new sonic fiber, based on phononic crystals, which has been enabled to couple environmental acoustic waves to the fiber core. Coupling the environmental acoustic waves to the fiber core may be applicable in electrical energy harvesting field. The proposed structure included a conventional phononic crystal fiber with a conical input, where solid rods where shaped in a conical wat at the input of the fiber. In this way, environmental acoustic waves can be properly coupled to the core region of the fiber. Acoustic wave leakage to the outside in the coupler part has been extremely decreased in comparison with initial coupling idea. Experimental results were shown that coupling the environmental acoustic waves to the core region without leakage by conical input part of proposed phononic crystal fiber is realizable. Electrical output of phononic crystal fiber is equal to 1.8 mV whereas this is 0.8 mV in bare case. This electrical output multiplication is almost equal to input area to the core area. This experimental result indicates that acoustic wave leakage to the outside is ignorable. Using the conical input technique in the phononic crystal fiber instead of external acoustic wave couplers such as acoustic lenses, reduces time and cost. Acoustic lenses may also have fabrication tolerances which adds an additional error to the system and reduces the accuracy of the work.

## Data Availability

The data that support the findings of this study are available from the corresponding author upon reasonable request.

## References

[1] Khelif A, Adibi A (2015). Phononic Crystals: Fundamentals and Applications.

[2] Khelif A, Choujaa A, Benchabane S, Djafari-Rouhani B, Laude V (2004). Guiding and bending of acoustic waves in highly confined phononic crystal waveguides. Appl. Phys. Lett..

[3] Pennec Y, Djafari-Rouhani B, Vasseur JO, Khelif A, Deymier PA (2004). Tunable filtering and demultiplexing in phononic crystals with hollow cylinders. Phys. Rev. E..

[4] Motaei F, Bahrami A (2020). Eight-channel acoustic demultiplexer based on solid–fluid phononic crystals with hollow cylinders. Photon. Nanostruct.: Fundam. Appl..

[5] Motaei F, Bahrami A (2020). Nonlinear elastic switch based on solid–solid phononic crystals. J. Mater. Sci..

[6] Lucklum R, Ke M, Zubtsov M (2012). Two-dimensional phononic crystal sensor based on a cavity mode. Sens. Actuators B Chem..

[7] Gharibi H, Bahrami A (2020). Phononic crystals for sensing FAMEs with demultiplexed frequencies. J. Mol. Liq..

[8] Tol S, Degertekin FL, Erturk A (2016). Gradient-index phononic crystal lens-based enhancement of elastic wave energy harvesting. Appl. Phys. Lett..

[9] Motaei F, Bahrami A (2022). Energy harvesting from sonic noises by phononic crystal fibers. Sci. Rep..

[10] Motaei F, Bahrami A (2021). An elastic fiber based on phononic crystals. Sci. Rep..

[11] Tol, S., Degertekin, F. L. & Erturk, A. Phononic crystal Luneburg lens for omnidirectional elastic wave focusing and energy harvesting. *Appl. Phys. Lett.* **111(1)**, (2017).

[12] Tol S, Degertekin FL, Erturk A (2019). 3D-printed phononic crystal lens for elastic wave focusing and energy harvesting. Add. Manuf..

[13] Hyun J, Park CS, Chang J, Cho WH, Kim M (2020). Gradient-index phononic crystals for omnidirectional acoustic wave focusing and energy harvesting. Appl. Phys. Lett..

[14] Watcharakitchakorn, O. & Silapunt, R. Design and modeling of the photonic crystal waveguide structure for heat-assisted magnetic recording. *Adv. Mater. Sci. Eng.* (2018).

